# Fertility Control and the Welfare of Free-Roaming Horses and Burros on U.S. Public Lands: The Need for an Ethical Framing

**DOI:** 10.3390/ani12192656

**Published:** 2022-10-03

**Authors:** Allen T. Rutberg, John W. Turner, Karen Herman

**Affiliations:** 1 Center for Animals and Public Policy, Cummings School of Veterinary Medicine at Tufts University, 200 Westboro Road, North Grafton, MA 01536, USA; 2 Department of Physiology and Pharmacology, University of Toledo, Toledo, OH 43606, USA; 3 Sky Mountain Wild Horse Sanctuary, P.O. Box 2946, Santa Fe, NM 87504, USA

**Keywords:** wild horse management, feral horse management, wild horse contraception, wildlife fertility control, wildlife contraception, public lands management, conservation ethics, wildlife ethics

## Abstract

**Simple Summary:**

Because the ethical foundation for the management of free-roaming horses and burros is very muddled, debates over approaches to their management, including fertility control, focus almost exclusively on practicality. In the U.S., this tendency is reinforced by the federal laws that govern public lands management, which center decision-making processes on calculations about how to divide yield among users. Inevitably, outside market forces bias these decisions towards overuse, which ultimately harms the land. In our view, the land and all its inhabitants, including free-roaming horses and burros, wildlife, and people, will only thrive if the pressures of commercial use are removed, and if management is guided by ethics of care, which have rich and diverse cultural origins. Application of care ethics will specifically provide more clarity to horse and burro management, including the selection of appropriate fertility control agents.

**Abstract:**

To be effective and publicly acceptable, management of free-roaming horses and burros in the United States and elsewhere needs a consistent ethical framing of the animals and the land they occupy. In the U.S., the two laws that largely govern wild horse and burro management, the 1971 Wild Free-Roaming Horse and Burro Act and the 1976 Federal Land Policy and Management Act (“FLPMA”), rest on conflicting foundations, the former based on an ethic of care and the latter on largely utilitarian principles. These conflicts specifically fuel debates over the selection of appropriate fertility control agents for horse and burro management. Because land-use and management decisions are largely controlled by the FLPMA, and because the ethical treatment of animals is typically considered under conditions established by their use, both the larger debate about equids and land management and the specific debate about fertility control are dominated by cost/benefit calculations and avoid broader ethical considerations. In our view, the long-term health and ethical treatment of free-roaming horses and burros, the lands they occupy, and the wildlife and people they share it with will require the replacement of the resource-use model with a more holistic, care-based approach.

## 1. Introduction

In 1991, Kirkpatrick and Turner [1] proposed a set of criteria for the “ideal wildlife contraceptive” (Table 1). These criteria have been echoed, critiqued, and revised in subsequent papers [2,3,4,5]. Many of their criteria are basically practical—safety, effectiveness, and low cost evoke pragmatic issues—but the criteria go beyond practicality to address the well-being of the animal. Remote delivery is more practical, reducing cost, time, and effort, but is also better for the animal because it reduces or eliminates stresses and risks associated with handling. That the ideal wildlife contraceptive should also minimize effects upon individual and social behaviors centers the ethical focus on the animal, implying that the target animal, and those around it, should be able to live their lives the way they would choose given the environments in which they live. 

Any contraceptive, of course, necessarily alters the behavior and choices available to and practiced by an animal that would otherwise bear offspring [5]. Females with offspring behave differently from females without offspring; but the ideal wildlife contraceptive should not add to those unavoidable constraints and alterations.

The Kirkpatrick and Turner criteria also support ethical principles that go beyond individual animal welfare. In referencing the food chain, they address the health of the environment, and potential risks to or physiological effects on predators and scavengers (including human ones). The stipulation that fertility control should be reversible means that use of the agent requires planning for more than one treatment cycle. However, reversible contraceptives are more protective of natural evolutionary processes and offer the treated individual a chance to return to normal reproductive activities and behaviors.

In developing these criteria, Kirkpatrick and Turner centered their thinking on free-roaming horses and burros, the animals on which they had focused their work up to that time. Their framing yielded a set of criteria that added animal- and eco-centric ethical criteria to purely human-centered utilitarian criteria, which focus on providing the most cost-effective solution to a human problem linked to animal population levels. 

This was, and remains, an unconventional approach to wildlife fertility control, and to animal welfare generally. Although there is much debate over how best to define and measure welfare [6,7], public and institutional views of what constitutes good welfare for animals most commonly depend on the contexts in which people use or do not use the animals, as well as on the ethical systems through which we frame these contexts and uses. Typically, assessments of animal welfare are framed in a utilitarian context in which costs and benefits to the human users are weighed against measures of animal welfare under conditions that are tightly constrained by the use [6,8]. Measures and assessments of welfare of animals raised for food, such as the Five Freedoms, the Five Domains, and their modifications, are constrained by and incorporate the users’ needs for their animals to survive, grow rapidly, produce many young, and not spread disease while keeping production costs down [6,9]. The welfare of laboratory animals often is defined and defended in the context of their reliability as models for biological processes and the need to carefully control the conditions under which they live [10]. At one extreme, society can be completely indifferent to the welfare of the animals we label as pests, and no method of killing them—glue traps, slow-acting poisons, snares, aerial shooting—is deemed too cruel [11,12]. At the other extreme, we frame even our best-cared for companion animals as members of human families, and many of our notions of their welfare flow from that concept. All these definitions of welfare are human-centric, constructed around and filtered through human needs and value systems that center people. 

Prevailing models of wildlife management likewise follow utilitarian production models of wildlife welfare. Wildlife ranches and other systems of private ownership of wildlife are overtly and explicitly production-focused. Under the North American Model of Wildlife Management, free-roaming wildlife are also construed as a resource, albeit one allocated democratically to the public under rules established by science to assure sustainability [13,14]. Under this model, wild animal populations (and by necessity the individuals that compose them) are considered “healthy” when they grow fast, reproduce quickly and abundantly, and survive well and disease-free until time of harvest by the public. Free-roaming wildlife are “taken” by methods “that inflict no unnecessary pain or suffering on game” [14], but what is considered “unnecessary” varies widely with the specific harvesting practice. Firearms hunters aspire to drop their targets immediately with a single shot, archery hunters expect a delay and a ”blood trail” before their target succumbs, and time from capture to death in animals subject to trapping can vary from minutes to days, depending in part on what kinds of traps are allowed, whether minimum trap-check times are set by state or other regulations, and whether in practice trappers comply with these regulations [12,15]. As noted, in free-roaming wildlife (native or non-native) characterized as pests, even the lax welfare standards applied to game species may be lifted.

Thus, however measured or assessed, animal welfare standards are highly labile when developed for animals that are framed as resources or otherwise deployed for human purposes. Within utilitarian framings of free-roaming horses and burros, we should likewise expect only practical criteria to be applied to welfare standards for application of fertility control or other management tactics. 

It is comfortable and easy to focus on practical challenges associated with administering contraceptives to free-roaming horses and burros [4,16], and only a few studies have seriously considered the welfare consequences of fertility control in free-roaming equids and other wildlife [5,17,18]. However, when we evaluate the ethics behind the application of contraceptives to these animals, we cannot ignore the context, the deeper questions about how we think about free-roaming horses and burros, and their place on the land. 

## 2. The Ethical Framing of U.S. Wild Horse and Burro Management Policies

The treatment of free-roaming horses and burros on U.S. public lands offers a particularly fertile test case for thinking about the role of framing in establishing animal welfare standards. Currently, approximately 86,000 free-roaming horses and burros occupy 10.9 million ha of public lands managed by the U.S. Bureau of Land Management (BLM)—about 11% of total BLM-owned lands [19]. Historically, management of horse and burro numbers using gathers, removals, and adoptions has been unable to keep up with population growth; at time of this writing (September 2022), 63,922 horses and burros occupy corrals and off-range pastures supported by U.S. taxpayers [20]. Thus, there has long been interest in employing fertility control to reduce population growth and bring removals more into line with demand for adoptions. 

Yet everyone frames horses and burros differently, and views only diversify when those animals are free-roaming [21,22,23]. Horses and burros are variously characterized as livestock, work animals, companion animals, “equine athletes”, and more; when free-roaming, they are additionally framed as pests, invasive non-native species, and wildlife. How we frame both the equids themselves and the purpose of the land they occupy will shape welfare standards, and both are widely and vociferously contested in policy debates [24,25,26]. These contradictions and conflicts are embedded in U.S. federal laws governing public lands management. 

The Federal Land Policy and Management Act of 1976, “FLPMA” (43 U.S.C. 1701), dictates a somewhat self-contradictory but principally utilitarian model of the public lands. Whereas, in its declaration of policy, Congress acknowledges that “where appropriate, [managers] will preserve and protect public lands in their natural condition” (43 USC 1701(a)8), the heart of the policy process, land use planning, establishes as its first criterion that the agencies shall “use and observe the principles of multiple use and sustained yield…” (43 U.S.C. 102 (a)7; 43 USC 1712 (c)1). Thus, with its “multiple use” and “sustained yield” language, the FLPMA firmly embeds the public lands in a production framing. The public lands yield resources; the federal agencies that manage the lands, the Bureau of Land Management and the U.S. Forest Service, use science to figure out how much resource there is, and then follow the land use planning process to decide who receives how much. What share of production goes to cows? How much goes to wild horses or burros? How much goes to wildlife? What shares of other public lands resources are allocated to other human users, the timber, mining, and various energy-production industries? 

The utilitarian language of the FLPMA establishes the land as publicly-owned and managed ranchlands. Its yield of cattle and other commercial livestock enters the global commodity market and supplies income to public lands ranchers. The wildlife yield is harvested by the public under the North American model, which promotes sustainability by excluding most of the yield from commodification. 

The “yield” of horses and burros is rounded up, removed, then allocated to the public via adoption (at least nominally non-commercial) or, potentially, commercial sale. However, because the public opposes the sale, commodification, and potential slaughter of wild horses and burros, and has exercised its preferences through Congress [27], the commercial value of wild horses and burros and hence their framing as livestock are compromised. (Only a small proportion of horses and burros removed from the range is sold [19]). In the utilitarian framing of the FLPMA, animals such as free-roaming horses and burros that have little or no commercial value and that compete with commercially valuable livestock or wildlife “resources” slip into the category of “pests” [23]. 

Embedding free-roaming horses and burros in a utilitarian framing—livestock at best, or pests at worst—gives managers free rein to create welfare standards of convenience, needing only to exceed the lowest bar of public acceptability. Hence managers can rely on roundups and removals, which for equines sever long-standing social and family relationships and subject previously independent animals to more or less environmentally and socially impoverished lives in pastures and in stables—not to mention exposing them to the direct stresses associated with roundups and handling by unfamiliar humans [5,18]. Removed from their homes and families, the best they can hope for is the companionship of (maybe) congenial stablemates and caring humans. Managers can also pursue invasive fertility control techniques, such as surgical sterilization, which may threaten life and health, dramatically alter social behavior and social relationships, deprive individuals of all future prospects for reproduction, and compromise local gene pools by narrowing the range of potential contributors. Applying these tactics grievously injures the welfare of horses and burros and the sustainability of their populations.

In stark contrast, the original 1971 Free-Roaming Wild Horse and Burro Act, which set aside designated federal lands for the protection of free-roaming horses and burros, largely frames free-roaming horses and burros as wild, but subject to management. “The Secretary shall manage wild free-roaming horses and burros in a manner that is designed to achieve and maintain a thriving natural ecological balance on the public lands” (16 U.S.C. 1333(a)). The passage goes on to say, “…all management activities shall be at the minimal feasible level…”, and ties management to “the natural ecological balance of all wildlife species.” Created as it was from an ethic of care for animals that were subject to abuse, and in the same era that produced the Endangered Species Act, the Act both respects the wild lives of free-roaming horses and burros and ties their management to the health of the land they occupy.

Raising the ethical bar for application of fertility control to wild horses and burros and for all management actions demands a different conception of their place on the land, and of the land itself, one more closely aligned with the vision of the 1971 Act.

## 3. Beyond Utilitarian Ethics for Wild Horses and Burros on the Public Lands

The FLPMA production model of the public lands unequivocally compromises the vision of the 1971 Act. Setting up the agencies as mediators of endless conflict among users, many of whom are driven by commercial and market interests extrinsic to the needs of the land itself, the FLPMA has failed to protect either the welfare of wild horses and burros or the “thriving natural ecological balance” of the land they occupy. This problem is likely to worsen with climate change [28]. The lesson of the North American Model of Wildlife Management holds: restoring wildlife to the landscape requires ending its commercialization and commodification [13,14]. To survive and thrive, the public lands and their inhabitants must be freed of their utilitarian framing, and in particular the utilitarian framing that supports their commodification.

What alternative framings are available for public lands?

One such conception of the public land is as “wilderness” [25,29]. The wilderness concept implies that human intervention, including application of fertility control to wild horses and burros (or, depending on your viewpoint, even their very presence) spoils the landscape and is undesirable, unethical, even sacrilegious [30,31]. The concept of wilderness is very appealing and is foundational to the conservation movement, but historical scholarship and the voices of the Indigenous people who were displaced and ignored to create both the concept and the legal designation of “wilderness” show clearly that a “pristine” North American landscape “discovered” by European settlers is and always was a fiction [31,32,33,34,35]. The wilderness concept relies on erasing ancient familial relationships and interdependencies between Indigenous people, the land, and its non-human inhabitants (including horses), and in doing so is both unethical and empirically wrong. 

Preferable to a utilitarian approach or a hands-off approach, we believe, is one founded in an ethic of care—as the 1971 Act is. Multiple authors, expressing both Indigenous and Western perspectives, have developed and applied ethics of care and familial connection to animals and to land [36,37,38,39,40,41,42]. The land occupied by wild horses and burros is shared with people and a multitude of other animals and plants. Engagement and mindful intervention in the lives of its equine occupants may sometimes be the right thing to do. With the application of fertility control, and all else, our approach should be guided by the ethical imperative to care for the land and all its inhabitants. 

We believe that under an ethic of care, what we owe wild horses and burros, and indeed all the land’s other wild inhabitants, are rich environments that offer choices that each animal can pursue consistent with its capacities and inclinations. This assertion has a number of implications. 

First, autonomy is crucial; moreover, it is dependent on the quality of the animal’s environment. An impoverished biological community, where forage is sparse, or movements are limited by restrictive fencing or by drought-driven need to stick close to a dwindling water hole, offers fewer choices and hence diminished autonomy. So does an impoverished social environment, where options for association and the development of long-lasting and dynamic social relationships are limited. Under these environmental conditions, concerns of autonomy and of welfare [5,18] are mutually reinforcing. 

Second, reducing horse and burro numbers by removals may be needed to protect the overall richness of the environment and the well-being of all animals remaining on the range, but it is a last resort. An animal-centered ethic cannot ignore the price paid by animals removed from the range, and we owe them the richest life possible should it be necessary. 

Third, any application of fertility control exerts a social and behavioral cost on animals that receive it, as well as on herdmates [5]. The beneficial trade-off lies in the protection of the richness of the community as it is experienced by all its inhabitants, and in the reduction in risk of removal and the potential social and environmental deprivation that goes with it. 

## 4. Conclusions

Although we must be humble when acting on what we think free-roaming horses and burros want, it is probably fair to infer that as social animals grounded in their experience of each other and their environment, they would prefer to stay at home, on the range, with their bands. Moreover, their choices are expanded when they live on a diverse and healthy rangeland. Judicious and effective application of minimally invasive fertility control can keep wild horses and burros at home, living rich lives with opportunities for choice on a landscape they share with other wildlife and the human animal too.

## Figures and Tables

**Table 1:** Kirkpatrick and Turner’s characteristics of an ideal wildlife contraceptive [1].

Contraceptive effectiveness of at least 90%
The capacity for remote delivery, with no handling of animals
Reversibility of contraceptive effects (more important for some species than others)
Safe to use in pregnant animals
Absence of significant health side-effects, short- or long-term
No passage of the contraceptive agent through the food chain
Minimal effects upon individual and social behaviors
Low cost

## Data Availability

Not applicable.
